# Ewing's sarcoma of the ulna treated with sub-total resection and reconstruction using a non-vascularized, autogenous fibular graft and hernia mesh: A case report

**DOI:** 10.3892/ol.2015.3534

**Published:** 2015-07-24

**Authors:** CONG WANG, NONG LIN

**Affiliations:** Department of Orthopaedic Surgery, Second Affiliated Hospital, School of Medicine, Zhejiang University, Hangzhou, Zhejiang 310009, P.R. China

**Keywords:** Ewing's sarcoma, forearm interosseous ligament, hernia mesh, non-vascularized fibular graft, ulna

## Abstract

Ewing's sarcoma of the bone is the second most frequently occurring malignant bone tumor in children and adolescents. Ewing's sarcoma in the ulna are extremely rare. Thus, the surgical options for reconstruction of the elbow are limited and technically challenging. In the current study, a 29-year-old male with Ewing's sarcoma of the ulna was treated with a sub-total resection and reconstruction using a non-vascularized, autogenous fibular graft and hernia mesh. At the 2-year follow-up, the patient had returned to his previous occupation with no evidence of local recurrence or distant metastasis. The functional recovery was satisfactory, and the patient could perform active movement of the elbow from 0° to 135°, forearm pronation to 30°, supination to 85° and had full hand function. The grip power of the left hand was 36 kg, which was 86% of the contralateral side (42 kg).

## Introduction

Ewing's sarcoma of the bone is the second most frequently occurring malignant bone tumor in children and adolescents. It is a member of the Ewing's sarcoma family of tumors, which also includes primitive neuroectodermal tumors, Ewing's soft tissue sarcomas and Askin's tumors. The Ewing's sarcoma family of tumors are high-grade aggressive lesions that most commonly originate in the bone, and are associated with large soft tissue masses and frequent metastases. The majority of Ewing's sarcomas of the bone are located in the lower extremities and pelvic girdle, but occasionally arise in the ulna (1). Previous treatments for Ewing's sarcomas, such as surgery (alone), radiotherapy or mono-chemotherapy, have failed to achieve ideal results. The majority of patients succumbed within two years, and the five-year survival rate was <20% (2). However, with progress in chemotherapy, the prognosis for patients with Ewing's sarcoma has improved considerably during the past three decades (3). Currently, chemotherapy and surgery are the standard treatment for Ewing's sarcomas (4). The ulna is an uncommon site for these malignant and aggressive tumors. Thus, the surgical options for reconstruction of the elbow are limited and technically challenging (4–8). The current study presents the case of a successful sub-total resection and reconstruction using a non-vascularized, autogenous fibular graft and hernia mesh in a 29-year-old male with Ewing's sarcoma of the ulna. Written informed consent was obtained from the patient.

## Case report

**Figure 1.**
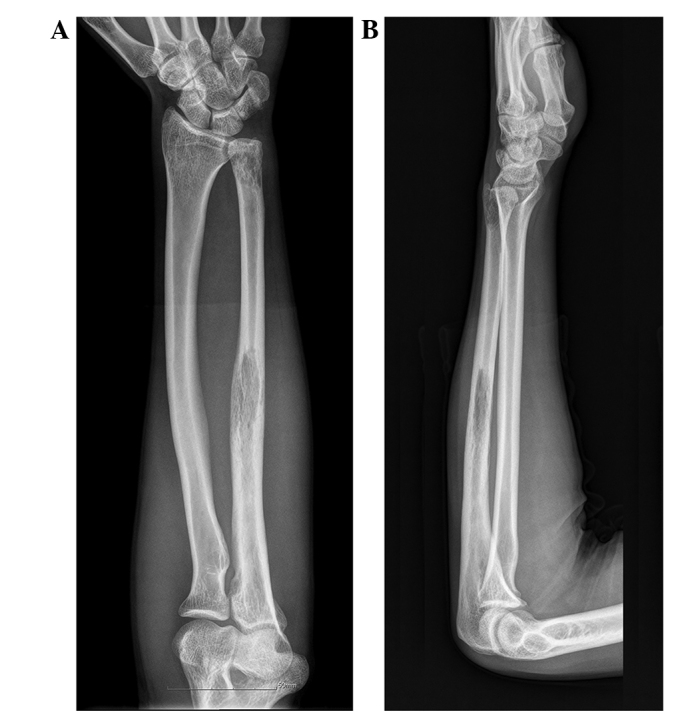
(A) Anteroposterior and (B) lateral plain radiographs showing an osteolytic lesion with cortical destruction in the proximal, middle and distal ulna.

**Figure 2.**
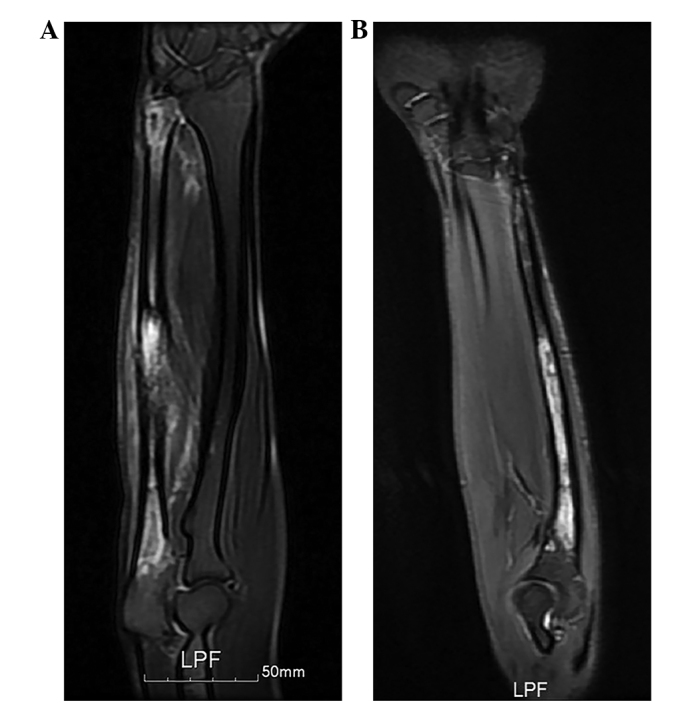
Magnetic resonance imaging findings (T2-weighted). (A) Prior to neoadjuvant chemotherapy. The intramedullary tumor involved nearly the full length of the ulna, with the exception of the proximal olecranon. The surrounding cortex was partially involved, and the soft-tissue components around the tumor appeared patchy and hyperintense. (B) Following neoadjuvant chemotherapy. The tumor regressed following two courses of intensive chemotherapy.

A 29-year-old male presented to the Second Affiliated Hospital (Hangzhou, Zhejiang, China) with a 3-month history of repeated left forearm pain. The initial consultation was performed on September 10, 2012. A plain radiograph demonstrated an osteolytic lesion with cortical destruction involving the proximal, middle and distal ulna (Fig. 1). Magnetic resonance imaging demonstrated an intramedullary tumor that involved nearly the full length of the ulna, with the exception of the proximal olecranon (Fig. 2). The surrounding cortex was partially involved, and the soft-tissue components around the tumor appeared patchy and hyperintense on T2-weighted images. The elbow and wrist joints were tumor free. The patient underwent an open biopsy, and frozen section examination demonstrated proliferation of small round cells with round-to-oval nuclei. Subsequently, immunohistochemistry revealed that the cell membrane was strongly positive for vimentin and cluster of differentiation 99. Thus, analysis of the tumor biopsy supported a diagnosis of Ewing's sarcoma. A bone scan revealed uptake only at the left ulna, and chest computed tomography showed no evidence of metastatic disease. Therefore, the patient was classified as having stage IIB according to the Enneking surgical staging system (5). On the following day, the patient received two 6-week courses of neoadjuvant chemotherapy, including 2.9 g ifosfamide (days 1–5 and 11–15), 150 mg cisplatin (day 22) and 95 mg doxorubicin (day 24). Restaging magnetic resonance imaging indicated tumor regression (Fig. 2), which confirmed the efficacy of the chemotherapy.

**Figure 3.**
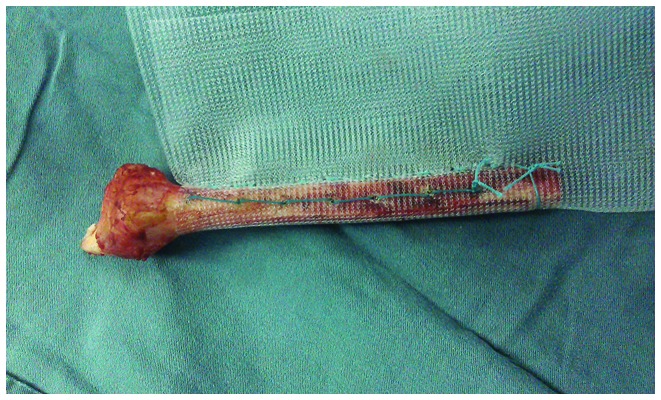
Intraoperative findings. The fibular graft was drilled and wrapped with hernia mesh.

**Figure 4.**
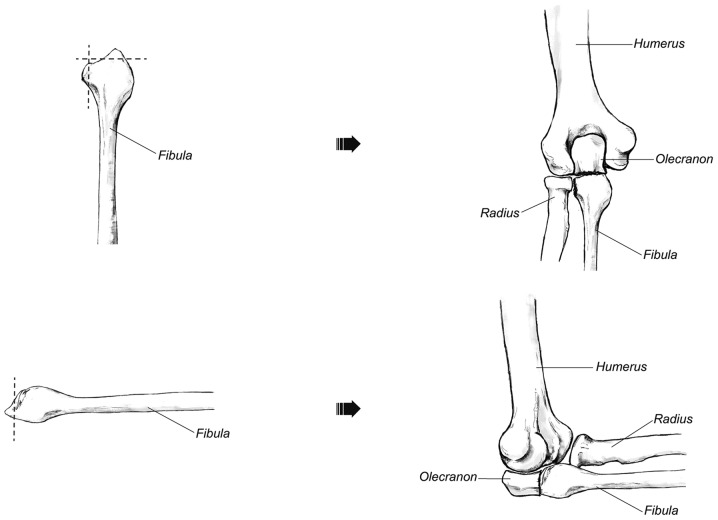
The fibular head was modified to match the remaining olecranon process and the articular surface at the ulnotrochlear and proximal radioulnar joints.

As the tumor involved nearly the full length of the ulna, the patient underwent a sub-total resection of the ulna (except for half of the olecranon process). Intraoperative frozen sections revealed a negative tumor margin. Reconstruction of the bony defect was performed using a non-vascularized, autologous, fibular graft harvested from the ipsilateral leg. The length of the harvested fibula, including the fibular head, was 15 cm. The fibular graft was drilled and wrapped with hernia mesh to recreate the forearm interosseous ligament (Fig. 3). Next, the mesh was sutured to the remnant interosseous ligament of the radius to hold the graft in position. The fibular head was modified to match the remaining olecranon process and the articular surface at the ulnotrochlear and proximal radioulnar joints to reconstruct the elbow joint (Fig. 4). This was fixed to the remnant olecranon process with a plate and screws. To stabilize the elbow joint, a Kirschner wire was used to fix the head of the radius and fibula. In addition, the medial collateral ligament and capsule of the elbow were fixed to the fibular head by an anchor. Post-operative chemotherapy was continued, and the Kirschner wire was removed 4 weeks after surgery. To protect the reconstructed elbow joint, 4 weeks of full immobilization with a gypsum cast at 90° flexion was applied after surgery, followed by an additional 4 weeks of passive- and active-assisted flexion and extension of the left elbow.

**Figure 5.**
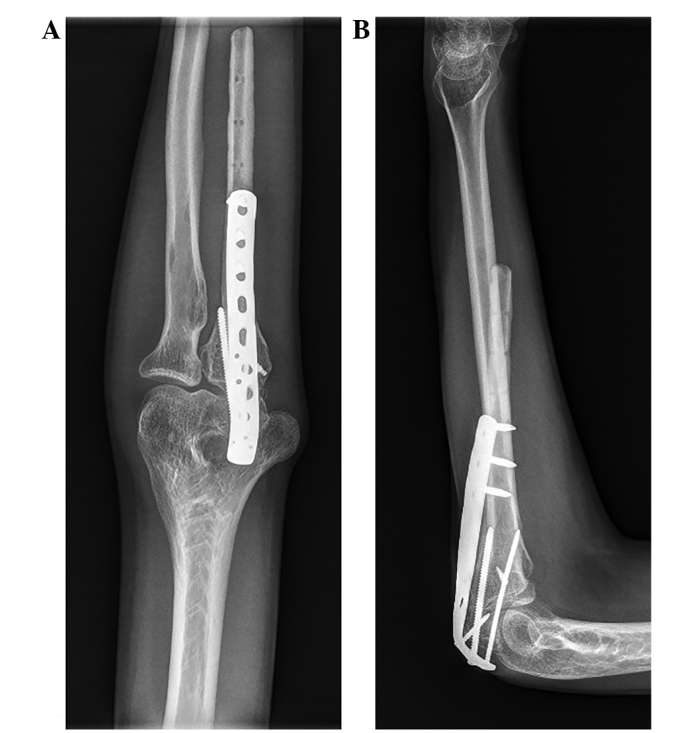
(A) Anteroposterior and (B) lateral radiographs 2 years after surgery. Restoration of the proximal ulnar anatomy occurred without severe problems.

**Figure 6.**
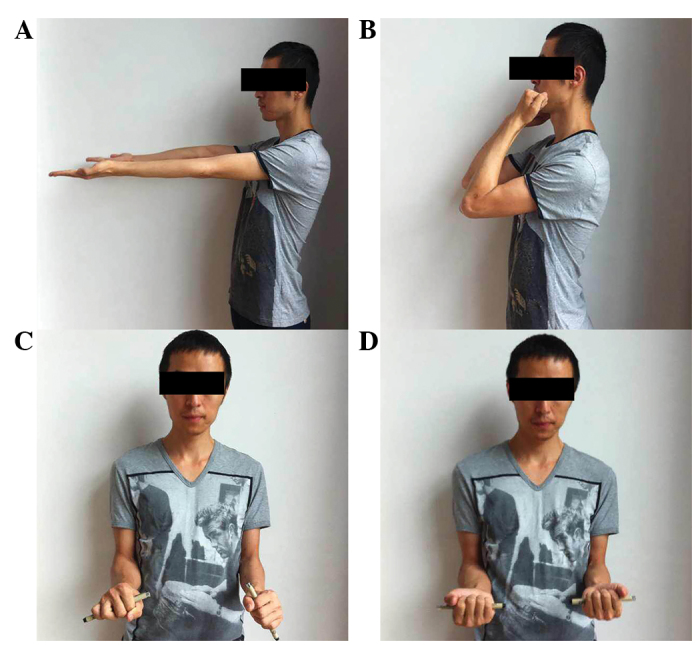
Images of arm (A) extension, (B) flexion, (C) pronation and (D) supination showing that the patient experienced excellent functional recovery 2 years after surgery.

At the 2-year follow-up, there was no evidence of local recurrence or distant metastasis (Fig. 5). The fibular graft was united with the olecranon proximally, and the functional recovery was satisfactory, with active movement of the elbow from 0° to 135°, forearm pronation to 30°, supination to 85° and full hand function (Fig. 6). The grip power of the left hand was 36 kg, which was 86% of the contralateral side (42 kg). No morbidity in the donor site was observed in the leg where the fibula was harvested and the functional capability of the leg was good. Furthermore, the patient had been able to return to his previous occupation.

## Discussion

Malignant and aggressive tumors located within the ulna are relatively rare, although there have been reports of osteosarcoma, primary giant cell tumors and Ewing's sarcoma of the ulna. Salvaging the limb following an ulnar tumor resection poses a complex, reconstructive challenge (6,7,9–11). Various options have been reported for the reconstruction of the elbow joint following ulnar tumor resections, such as radius neck-to-humerus trochlea transposition, endoprosthetic reconstruction, allografts, vascularized fibular grafts and extracorporeal irradiation (6,7,9,10). Due to the location of the tumors, previous studies primarily involved reconstructions of partial ulna defects, and the remnant ulna provided the basis for bone grafts or prosthesis implantation. However, malignant and aggressive tumors invading nearly the full length of the ulna have rarely been reported. Thus, the present patient presented a unique surgical challenge. Sułko (6) reported a patient with Ewing's sarcoma who underwent radius neck-to-humerus trochlea transposition following an extensive resection of the proximal portion of the ulna. The technique of radius neck-to-humerus trochlea transposition was a good candidate for reconstruction of the elbow joint following wide resection of the ulna. However, the functional rotation of the forearm may be unsatisfactory. Duncan *et al* (7) performed this procedure in 2 adult patients. One patient achieved 20° pronation, but no supination, and the second patient achieved 10° pronation and limited supination ability (9). Therefore, in the present study, it was proposed that the elbow joint would be reconstructed using a non-vascularized, autogenous fibular graft and hernia mesh. The functional recovery was satisfactory, particularly for functional forearm rotation. The patient achieved forearm pronation to 30° and supination to 85°.

In the present case, as a sub-total resection of the ulna was performed, disruption of the forearm interosseous membrane was inevitable. The forearm interosseous membrane is a dynamic and complex fibrous structure that maintains the stability of the longitudinal forearm and transmits forces between the radius and the ulna (8). To reconstruct the forearm interosseous membrane, a hernia mesh, which has biological properties to provide mechanical constraint, was applied in the present study. However, the purpose of reconstructing the forearm interosseous membrane was not only to restore its own function, but also to maintain the function of the forearm and elbow joint. This was very important in three ways. First, the mesh assisted in holding the fibular graft in position. Second, it repaired the connection between the radius and ulna to maintain the stability of the elbow joint; the stability of the elbow joint is highly dependent on the integrity of the interosseous membrane and the balance between the radius, ulna and humerus. Additionally, the hernia mesh may provide a biocompatible scaffold for soft tissue ingrowth. Third, it may improve active movement of the elbow and functional rotation of the forearm. Following a sub-total resection of the ulna, the flexor apparatus of the elbow was primarily dependent on the biceps tendon for normal movement; the reconstruction of the interosseous membrane can transmit forces from the radius to the ulna to drive elbow flexion. Similarly, the force transmission between the radius and ulna by the interosseous membrane can also aid in restoring the functional forearm rotation. This method of reconstruction of the forearm interosseous membrane has not been previously reported, but the use of synthetic mesh during limb salvage surgery is not rare. Marulanda *et al* (12) suggested that the application of a synthetic vascular mesh for a reconstruction of the proximal humerus may reduce the number of dislocations and aid in soft tissue attachment. In the present case, the elbow and forearm rotation was satisfactory, and no complications were observed.

Preservation of the proximal half of the olecranon during excision has been performed for the treatment of proximal ulna tumors. Goyal *et al* (11) reported a case of a desmoplastic fibroma of the proximal ulna that was treated with excision and reconstruction of the olecranon with a fibular graft. Bone-to-bone repair is often successful when a small region of the proximal olecranon may be preserved in continuity with the tendon insertion. This maintains a certain degree of integrity of the extensor apparatus of the elbow, resulting in useful elbow function and providing stability to the elbow joint. This technique was also adopted in the present study patient. To maintain stability in the elbow joint, a Kirschner wire was used to fix the fibular head and radius. Furthermore, an anchor was used to fix the medial collateral ligament, as its primary function is to resist elbow valgus overload.

Complications that could occur following reconstructive surgery include non-union, infection, joint degeneration and tumor recurrence. In the present patient, the non-vascularized, autogenous fibula was used as the bone graft. A free vascularized fibula may be a better choice for elbow reconstruction. For the irregular shape of the fibular head, it was hard to match the articular surface at the ulnotrochlear and proximal radioulnar joints. Therefore, the fibular head was modified to reconstruct the elbow joint. Moreover, a two-year follow-up may be insufficient, and a longer follow-up is required to focus on oncological surveillance and long-term functional outcomes.

Additionally, the distal end of the ulna was not reconstructed in the present case, as a previous study demonstrated that routine reconstruction of this bone defect is not necessary following en bloc resection of tumors of the distal end of the ulna (13). Furthermore, the current patient was satisfied with the function and appearance of the upper extremity.

In conclusion, Ewing's sarcoma of the ulna is rare and the surgical options for reconstruction are technically challenging. In the present case, a successful sub-total resection of the ulna and reconstruction using a non-vascularized, autogenous fibular graft and hernia mesh was performed. This case indicates that using hernia mesh to reconstruct the interosseous membrane of the forearm may maintain the stability of the elbow joint and improve functional rotation of the forearm.
